# Sugar levels relate to aggression in couples without supporting the glucose model of self-control

**DOI:** 10.3389/fpsyg.2014.00572

**Published:** 2014-06-10

**Authors:** Florian Lange, Robert Kurzban

**Affiliations:** ^1^Department of Neurology, Hannover Medical SchoolHannover, Germany; ^2^Department of Psychology, University of Pennsylvania, Philadelphia, PA, USA

**Keywords:** blood glucose, self-control, aggression, self-regulation, ego depletion

As researchers in the field of self-control, we read the recent publication by Bushman et al. (2014) with great interest. Using creative measures of aggressive tendencies, the authors examined the relationship between blood glucose levels and proxies for intimate partner violence. Across 3 weeks of testing, daily measures of blood glucose appeared to be related to the number of needles participants stuck in a voodoo doll supposed to represent their partner. On a subsequent laboratory task, participants with low average glucose levels exposed their spouses to more aversive noise.

From their results, Bushman et al. (2014) concluded that glucose “influences aggressive tendencies and behaviors” (p. 3) within couples. They regarded their findings as implying that “interventions designed to provide individuals with metabolic energy might foster more harmonious couple interactions” (p. 3). While there is obvious appeal to the notion that glucose can increase self-control and thus prevent aggressive impulses from being expressed, this study does not provide evidence supporting this idea.

The work by Bushman et al. draws on the proposal that “self-control requires brain food in the form of glucose” (p. 3). However, the glucose model of self-control (Gailliot et al., 2007) suffers from both conceptual shortcomings and empirical falsification (Kurzban et al., 2013). Not only has the proposal that glucose fuels the part of the brain needed to exert self-control been shown to be inconsistent with what is known about brain metabolism (Kurzban, 2010), but the empirical evidence reported in support of the proposal has been demonstrated to be implausible from a statistical perspective (Schimmack, 2012). Note that the original multi-study report by Gailliot et al. (2007) argued that self-control depletion is mediated by blood glucose levels on the basis of nine studies, all of which produced significant results. Across these studies, effect sizes were strongly negatively correlated with sample sizes, and the probability of obtaining the observed pattern of only significant results was less than 1%, (Schimmack, 2012), implying that the role of blood glucose in determining self-control capacities is likely to be overstated. This conclusion is further corroborated by replication studies that did not find the originally reported effect (Lange and Eggert, 2014; Lange et al., in press) as well as additional work suggesting that rinsing one’s mouth with glucose is sufficient to counteract self-control fatigue (Molden et al., 2012; Sanders et al., 2012; Hagger and Chatzisarantis, 2013; see also Carter and McCullough, 2013).

In view of these issues, self-control and blood glucose levels cannot simply be equated. As a consequence, when relating their outcome measure to blood sugar concentrations, Bushman et al. (2014) did not test, as they claim, “the effects of self-control on aggression” (p. 3). What they did test was the size of the relationship between daily fluctuations in blood glucose levels and a measure of aggressive impulse. Importantly, the authors did not record any self-control data and assuming that the number of pins stuck in a doll varies according to individuals’ ability to exert self-control is conceptually problematic. For the daily assessment of aggressive tendencies, participants were simply asked to indicate how angry they were with their partner. They were not required to inhibit or override their aggressive thoughts, emotions, or urges. Hence, the only conclusion licensed by the findings reported by Bushman et al. is that blood glucose relates to a single-item self-report measure of aggressive impulse, not to the ability to control these impulses. We do not doubt that hungrier organisms are more aggressive. This accords with our everyday experience, the animal literature (e.g., Cook et al., 2000), and the Snickers ad campaign, “You’re Not You When You’re Hungry.” However, this observation does not imply that glucose reflects the fuel necessary to muster the willpower not to harm one’s partner.

For their second analysis, mean blood glucose levels across 3 weeks were related to aggressive behavior toward the partner. Analyzed in this way, glucose levels do not indicate the current state of a fluctuating self-control resource, but are rather a trait variable. This has important implications for the authors’ conclusions. The more aggressive participants on the laboratory task were not those who were ego-depleted or hungry in that particular moment. They had low blood sugar concentration in general, a trait that can be linked to aggression via numerous third variables. With increasing age, for instance, mean blood glucose levels increase (Dahle et al., 2009), while aggression diminishes (McLaughlin et al., 1992; Barbaree et al., 2003). Whereas the reported correlation might provide information about the biology of individual differences in aggression, it does not support the glucose model of self-control.

Experimental studies manipulating participants’ blood sugar concentration might provide evidence for the claim that “glucose levels are an important influence on self-control and aggression” (p. 3). To date, however, experimental evidence is insufficient to support a significant role of glucose in self-control fatigue (Kurzban et al., 2013). Likewise, Bushman et al.’s study does not demonstrate that fluctuations in blood glucose affect individuals’ self-control abilities. As an important consequence, there is no reason to assume that giving couples a sugary “boost to their self-control energy” (p. 3) will reduce intimate partner violence. Because the glucose model of self-control lacks empirical foundation, it does not qualify as a framework for scientifically based intervention strategies.

## Conflict of interest statement

The authors declare that the research was conducted in the absence of any commercial or financial relationships that could be construed as a potential conflict of interest.
